# Metabolite Contents and Antioxidant Activities of Soybean (*Glycine max* (L.) Merrill) Seeds of Different Seed Coat Colors

**DOI:** 10.3390/antiox10081210

**Published:** 2021-07-28

**Authors:** Yu-Mi Choi, Hyemyeong Yoon, Myoung-Jae Shin, Yoonjung Lee, On Sook Hur, Bong Choon Lee, Bo-Keun Ha, Xiaohan Wang, Kebede Taye Desta

**Affiliations:** 1 National Agrobiodiversity Center, National Institute of Agricultural Sciences, Rural Development Administration, Jeonju 54874, Korea; ymchoi@rda.go.kr (Y.-M.C.); mmihm@korea.kr (H.Y.); smj1204@rda.go.kr (M.-J.S.); yoon112@rda.go.kr (Y.L.); oshur09@rda.go.kr (O.S.H.); a649987318@korea.kr (X.W.); 2 Crop Foundation Division, National Institute of Crop Science, Rural Development Administration, Wanju 55365, Korea; bclee@rda.go.kr; 3 Division of Plant Biotechnology, Chonnam National University, Gwangju 61186, Korea; bkha@jnu.ac.kr; 4 Department of Applied Chemistry, Adama Science and Technology University, Adama 1888, Ethiopia

**Keywords:** antioxidant activity, fatty acids, *Glycine max*, oil, phenolic content, protein, seed coat color, soybean

## Abstract

Seed coat color is one of the main agronomical traits that determine the chemical quality of soybean seeds and has been used as a parameter during cultivar development. In this study, seeds of yellow (*n* = 10), greenish-yellow (*n* = 5), and light-yellow (*n* = 4) soybean accessions were evaluated for their contents of total protein, total oil, total phenolic (TPC), and five prominent fatty acids including palmitic acid (PA), stearic acid (SA), oleic acid (OA), linoleic acid (LA), and linolenic acid (LLA), relative to a control cultivar, and the effect of seed coat color on each was investigated. Antioxidant activity was also evaluated using 1,1-diphenyl-1-picrylhydrazyl (DPPH) radical scavenging activity, Trolox equivalent antioxidant capacity (TEAC), and ferric reducing antioxidant power (FRAP). The results showed significant variations of metabolite contents and antioxidant activities between the soybeans. The average TPC, DPPH-radical scavenging activity, and FRAP were each in the order of greenish-yellow > yellow > light-yellow soybeans. In contrast, light-yellow soybeans contained a high level of OA and low levels of SA, LA, and LLA, each except LA differing significantly from yellow and greenish-yellow soybeans (*p* < 0.05). Our findings suggest that greenish-yellow and light-yellow soybeans could be good sources of antioxidants and high-quality soybean oil, respectively.

## 1. Introduction

Soybean seeds are rich sources of health-promoting metabolites such as phenolic acids, isoflavones, and anthocyanins. These polyphenolic compounds are one of the natural antioxidants and have been used as supplements in food formulations on account of their role in preventing many ailments, including cancer, diabetes, and cardiovascular diseases [1,2,3]. Soybean seeds are also excellent sources of plant-based and high-quality protein and oil. Earlier studies pointed out that the protein and oil contents in soybean seeds may account for 35 to 55% and 13 to 26% on a dry seed weight basis, respectively [4,5]. It is widely documented that soybean protein contains all the essential amino acids. Owing to these, soybean has been used in the human diet in a variety of forms such as infant formula, protein isolates, and flours [6,7]. On the other hand, five fatty acids including two saturated fatty acids (palmitic acid (16:0) and stearic acid (18:0)) and three unsaturated fatty acids (oleic acid (18:1), linoleic acid (18:2), and linolenic acid (18:3)) [8,9] are predominantly found in soybean oil and have various health benefits. Overall, soybean oil has become a dietary choice of many societies around the world, partly because its consumption is associated with a reduced risk of cardiovascular diseases. Besides its dietary role for humans, soybean oil is becoming a major feedstock for biodiesel production. In 2019 alone, 77% of the total biodiesel production was derived from vegetable oils and out of that soybean oil contributed 30% [10]. Because of these, researchers have been modifying the levels of specific fatty acids to enhance the performance of soybean oil in biofuel blends [11]. In general, polyphenolic compounds, protein, oil, and fatty acids are among the key driving factors for the increasing application of soybean in the food, pharmaceutical, and biofuel industries.

The metabolite contents of soybean seeds, in general, are affected by both environmental and genetic factors. Temperature, growing location, cultivation year, farming condition, and solar radiation are among the major environmental factors and much work has been conducted by different researchers on the connection between these factors and soybean metabolites [7,12,13]. From a genetic standpoint, seed-related properties such as seed coat color, seed weight, and maturity are the key agronomical characteristics that determine soybean seed quality. Soybean seeds are found in various forms of seed coat colors such as black, yellow, brown, reddish, and green. Although most soybean varieties possess unicolored seeds, bicolor soybean seeds that possess a combination of two colors are also available [14]. Researchers identified several minor and major genes that regulate these characters, which in turn affect the levels of chemical components in matured seeds [2,14,15]. Some studies also documented the influence of seed coat color on the levels of secondary metabolites including isoflavones, anthocyanins, and tocopherols among others in soybeans [13,16,17,18]. In contrast, the effects of seed-related characteristics on protein, oil, and fatty acid contents were poorly investigated [4,9,19,20]. In general, analyzing the effects of both genetic and environmental factors on metabolite contents in soybean seeds is useful to identify desirable characteristics that lift consumers’ preferences [21]. In another aspect, unfavorable weather conditions triggered by global warming are challenging the production of high-quality soybeans. Therefore, such studies are consistently required since they provide vital information during the development of better-quality soybean cultivars [14].

In Korea, there is a growing interest and attention to develop improved soybean cultivars to meet the increasing demand. Previously, several researchers assessed the levels of various classes of metabolites in soybean genotypes cultivated in Korea. Besides, the effects of some environmental and genetic factors were investigated, though such studies are infrequent [4,9,13,22]. As part of ongoing research to identify high-quality soybean genotypes for future farming, this study aimed to comparatively evaluate the contents of several metabolites including total protein, total oil, five fatty acids (palmitic acid, stearic acid, oleic acid, linoleic acid, and linolenic acid) and total phenolic contents, and antioxidant activities including 1,1-diphenyl-1-picrylhydrazyl (DPPH) radical scavenging activity, Trolox equivalent antioxidant capacity (TEAC), and ferric reducing power (FRAP) in the seeds of yellow, greenish-yellow, and light-yellow soybean accessions relative to a control cultivar and assess the influence of seed coat color on each.

## 2. Materials and Methods

### 2.1. Chemicals and Reagents

All the chemicals and reagents used in this study were of analytical grade and were used without further purification. Water and *n*-hexane were purchased from Thomas Scientific (Philadelphia, PA, USA) and Fisher Scientific (Pittsburgh, PA, USA), respectively. All the other chemicals and reagents including methanol, anhydrous sodium sulfate (Na_2_SO_4_), sulfuric acid (H_2_SO_4_), boron trifluoride-methanol (BF_3_-MeOH) solution, sodium hydroxide (NaOH), gallic acid, Folin-Ciocalteu phenol reagent, *L*-ascorbic acid, sodium carbonate, potassium ferricyanide, trichloroacetic acid, ferric chloride, diammonium salt of 2,2′-azino-bis(3-ethylbenzothiazoline-6-sulfonic acid) (ABTS), 1,1-diphenyl-2-picrylhydrazyl (DPPH) radical, and 6-hydroxy-2,5,7,8-tetramethylchroman-2-carboxylic acid (Trolox), and standards of palmitic acid, stearic acid, oleic acid, linoleic acid, and linolenic acid were obtained from Sigma Aldrich (St. Louis, MO, USA).

### 2.2. Soybean Cultivation and Sample Preparation

**Figure 1:**
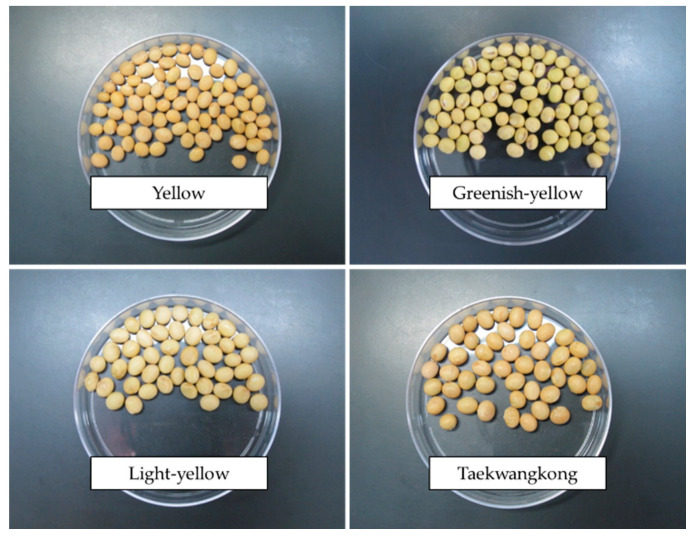
Seed samples of soybeans of different seed coat colors and a control cultivar.

The seeds of 19 soybean genetic resources, each having a specific introduction number, were obtained from the gene bank of the National Agrobiodiversity Center, Rural Development Administration (Jeonju, Korea). The seeds were sown on 4 June 2019, in an experimental field at the center and cultivated under similar environmental conditions [23]. Taekwangkong, a commercial soybean cultivar with yellow seed coat color, was similarly cultivated and used as a control cultivar. Matured soybean seeds were harvested and grouped as yellow (*n* = 10), greenish-yellow (*n* = 5), and light-yellow (also called pale-yellow) (*n* = 4) based on their seed coat color (Figure 1). Analysis of metabolite contents and antioxidant activities was determined using the whole seed samples of each soybean. Initially, the soybean seeds were dried in an oven (Bionex Convection, Vision Scientific, Daejeon, Korea) for three days at 50 °C and powdered using an electronic grinder. The powder of each sample was passed through a 315 μm sieve and made ready for extraction. Powdered seed samples were stored at −20 °C when not used. For ease of presentation, the soybean accessions were coded based on their entrance into their respective group. Information about the studied soybeans including name, introduction number, seed coat color, and code is provided in Table 1.

### 2.3. Determination of Total Protein Content

The total protein content was determined according to the Kjeldahl method using a Kjeltec instrument equipped with an auto-digester (FOSS, Tecator, Hoganas, Sweden) [4]. In brief, 0.5 g of powdered whole seed sample was placed into a digestion tube followed by the addition of 12 mL of concentrated H_2_SO_4_ and two pellets of selenium catalyst. The digestion process lasted for 1 h and the tube was then removed and cooled at room temperature (~25 °C). The final cooled solution was processed using an automatic Kjeltec analyzer (FOSS, Tecator, Hoganas, Sweden) which was capable of distillation and colorimetric titration. The total protein content (%) was auto-computed as:

(1) N×6.25

where *N* and 6.25 represent the released nitrogen content and a standard conversion factor, respectively.

### 2.4. Determination of Total Oil Content

A standard Soxhlet extraction procedure using a Soxtec extraction system (FOSS, Tecator, Hoganas, Sweden) was applied to estimate the total oil content of each soybean [24]. In brief, 0.7 g of powdered whole seed sample was placed in an extraction thimble followed by the addition of 50 mL of *n*-hexane. The thimble containing the mixture was loaded in an extraction unit maintained at 135 °C and the boiling, rinsing, and recovery times were automated at 30, 60, and 20 min, respectively. After extraction, the oil was cooled at 25 °C in a desiccator. The total oil content, as a percent, was determined from the mass ratio of the obtained oil to the extracted seed sample on a dry weight basis from triplicate repetitions.

### 2.5. Fatty Acid Analysis Using Gas Chromatography Coupled to Flame Ionization Detector

For fatty acid analysis, fatty acid methyl esters (FAMEs) were initially synthesized from the oil extracts through transmethylation using BF_3_-MeOH solution [25]. A QP2010 gas chromatography (GC) equipped with a flame ionization detector (FID) (Shimadzu, Kyoto, Japan) was used for structural analysis and quantification of FAMEs. During the sample run, 10 µL of FAMEs sample was injected into the GC-FID and separated using an HP-INNOWAX column (30 m × 0.250 mm × 0.25 µm). The temperature gradient of the column was optimized to start with 100 °C followed by an increase to 240 °C at a rate of 6.5 °C/min for 25 min. The temperatures of the injection port and the detector were each set at 250 °C. Helium was used as a carrier gas at a flow rate of 1.5 mL/min and a split ratio of 50:1. Individual FAMEs were identified using the corresponding standards and quantified as the percentage of the total fatty acid from peak areas of the obtained GC-chromatograms.

### 2.6. Total Phenolic Content and Antioxidant Activities

The total phenolic content (TPC) and antioxidant activities including 1,1-diphenyl-2-picrylhydrazyl (DPPH) radical scavenging activity, Trolox equivalent antioxidant capacity (TEAC), and ferric reducing antioxidant power (FRAP) were determined according to our recently reported method as described below [23].

#### 2.6.1. Extraction and Determination of TPC

Extraction of phenolic compounds was conducted using 70% acetone as a solvent. Initially, 1.0 g of powdered whole seed sample was mixed with 15 mL of the solvent in a 45 mL extraction tube. The mixture was sonicated at 25 °C in the dark for 25 min and the supernatant was recovered after centrifugation for 10 min at 4000 rpm. The residue was re-extracted one more time using 5 mL of the solvent. The total phenolic content (TPC) of each soybean was determined using the Folin-Ciocalteu method. In brief, 100 μL of the phenolic extract was reacted with an equal volume of Folin-Ciocalteu reagent at 25 °C in the dark. After 3 min, 100 μL of Na_2_CO_3_ (2%) solution was added, and the mixture was incubated for another 30 min. Finally, the absorbance was measured at 750 nm (Eon Microplate Spectrophotometer, Bio-Tek, Winooski, VT, USA), and the total phenolic content was computed as gallic acid equivalents (mg) per g of dried seed weight (mg GAE/g) from triplicate measurements.

#### 2.6.2. DPPH-Radical Scavenging Activity

To determine the DPPH-radical scavenging activity, 100 μL of phenolic extract of each soybean sample, in triplicate, was mixed with 100 μL of DPPH (150 μM) solution in the dark and the mixture was incubated for 30 min at 25 °C. Then, absorbance was measured at 517 nm (Eon Microplate Spectrophotometer, Bio-Tek, Winooski, VT, USA) and the DPPH radical scavenging activity was calculated as:

(2) $$\left(1-\left(\frac{A_{Sample}-A_{Blank\;sample}}{A_{Control}-A_{Blank\;control}}\right)\;\times \;100\right)$$

where *A* represents absorbance. An ascorbic acid solution at various concentration levels (1–100 mg/L) was used to plot a calibration curve, and the result was expressed as the ascorbic acid equivalent (mg) per g of dried seed weight (mg AAE/g).

#### 2.6.3. TEAC Assay

For the TEAC assay, ABTS·+ was initially prepared by mixing solutions of ABTS (7 mM) and potassium persulfate (K_2_S_2_O_8_, 2.45 mM) in the dark. The mixture was incubated for 16 h at 25 °C and diluted with water to an absorbance of 0.700 ± 0.02 at 734 nm. During analysis, 10 μL of the phenolic extract, in triplicate, was mixed with 150 μL of diluted ABTS·+ solution and the mixture was incubated at 25 °C in the dark. Then, the absorbance was measured at 734 nm (Eon Microplate Spectrophotometer, Bio-Tek, Winooski, VT, USA) against a blank solution, and the TEAC was calculated as Trolox equivalent (mg) per g of dried seed weight (mg TE/g).

#### 2.6.4. FRAP Assay

During the FRAP assay, 60 µL of the phenolic extract was placed in a 1.5 mL reaction tube. Then, 150 µL of freshly prepared phosphate buffer (pH: 6.6, 0.2 M) was added followed by the addition of an equal volume of 1% potassium ferricyanide (K_3_Fe(CN)_6_). After 20 min of incubation at 50 °C, 150 µL of 10% trichloroacetic acid was added and the mixture was centrifuged at 3000 rpm for 10 min. Then, 100 µL of the upper supernatant was sequentially diluted with 100 µL of distilled water and 20 µL of 0.1% ferric chloride solution. Finally, the solution was incubated for 10 min and absorbance was recorded at 700 nm (Eon Microplate Spectrophotometer, Bio-Tek, Winooski, VT, USA). The FRAP activity was expressed as ascorbic acid equivalent (mg) per g of dried seed weight (mg AAE/g). 

### 2.7. Data Analysis

During all the quantification procedures, measurements were made in triplicates, and results are expressed as the mean ± standard deviation (SD). In each of the soybean seeds, the total saturated fatty acid content (TSFA) was taken as the sum of palmitic acid and stearic acid, while the total unsaturated fatty acid content (TUFA) was determined as the sum of oleic acid, linoleic acid, and linolenic acid. A one-way analysis of variance was computed using the XLSTAT-software (Addinsoft, Long Island, NY, USA) and differences were considered as statistically significant at a probability value of < 0.05 (*p* < 0.05) based on Duncan’s multiple range tests unless specified. Principal component analysis (PCA), Ward’s hierarchical cluster analysis, boxplots, correlation analysis, and heatmaps were executed using the *R*-software *ver*. 4.0.2 (https://www.r-project.org/, accessed on 29 June 2021).

## 3. Results and Discussion

### 3.1. Total Protein and Total Oil Contents

Significant variations of total protein and total oil contents were observed between the soybean accessions, showing a wide genetic difference (*p* < 0.05). With a mean of 39.20%, the total protein content was in the range of 36.28–44.19%. Similarly, the total oil content ranged from 13.45 to 18.98% with a mean of 16.92% (Table 1). These ranges were comparable with the total protein (38.97–44.46%) and total oil (13.45–20.38%) contents previously reported in Korean soybeans [4]. In another study, Lee et al. [9] found a less wide but higher total protein content (46.01–48.44%), and a less wide total oil content (15.74–17.20%) compared to our results. Moreover, Kumar et al. [21] found total protein and total oil contents in the ranges of 32.20–42.10% and 15.40–22.00%, respectively in Indian soybeans. Relatively, more wide ranges of total protein (31.79–49.78%) and total oil (14.17–22.76%) contents were reported in Chinese soybeans [26]. These observations indicate the wide-ranging natures of both total protein and total oil contents in soybeans. In addition to the variation in the number of studied genetic resources, differences in genotypes, location, farming conditions, temperature, and year of cultivation could cause such differences [20,27].

Among the studied soybeans, accession GY14 contained the highest total protein and the lowest total oil contents, each differing significantly from the rest of the accessions (*p* < 0.05). The highest total oil content detected in GY12 was also significantly different from all the rest accessions except GY15 and LY16 (*p* < 0.05). These latter two accessions displayed the second and third highest total oil contents, respectively. As can be seen in Table 1, the soybean accessions had contrasting levels of total protein and total oil. This observation was expected since previous studies also noted similar trends and verified that several genes that impose pleiotropic effects on agronomical traits could cause such a reverse association [28,29,30]. Notably, many of the accessions outweighed taekwangkong, the control cultivar, at the level of total protein. Only two accessions including YL9 and GY15 had a significantly lower total protein content than that of taekwangkong (*p* < 0.05). In terms of total oil content, however, only two accessions (GY15 and LY16) displayed a significantly higher content than that of taekwangkong (*p* < 0.05). These accessions could be potential sources of a high level of soybean oil if considered in future farming.

**Figure 2:**
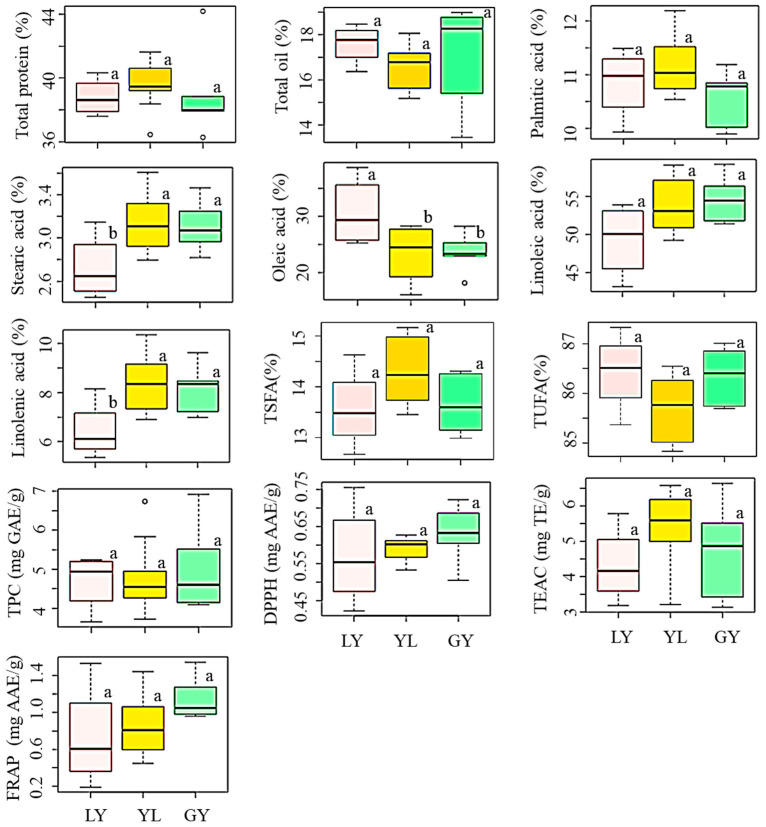
Variation of total protein, total oil, individual and total fatty acids, total phenolic content, and antioxidant activities in soybeans according to seed coat color. Different letters (a,b) on boxplots in a specific group represent the means that are significantly different (*p* < 0.05). TEAC: Trolox equivalent antioxidant capacity; DPPH: DPPH-radical scavenging activity; FRAP: Ferric reducing antioxidant power; GY: Greenish-yellow; LY: Light-yellow; TPC: Total phenolic content; TSFA: total saturated fatty acid; TUFA: Total unsaturated fatty acid; YL: Yellow.

We also investigated the influence of seed coat color on the levels of total protein and total oil (Figure 2, Table S1). The average total protein content was in the order of yellow > greenish-yellow > light-yellow soybeans while the average total oil content showed the opposite trend. Despite these differences, however, color variation did not bring a significant difference in any of the contents (*p* < 0.05). Yellow soybeans are widely cultivated worldwide, and several researchers analyzed their chemical compositions. Compared to our results, Cho et al. [4] and Lee et al. [9] previously reported slightly higher levels of total protein and total oil contents in yellow soybeans cultivated in Korea. It is interesting to note that these studies also failed to find significant variations in total protein and total oil contents in response to seed coat color differences which agreed with our results. In another study, Zarkadas et al. [31] noticed a small but significant difference in protein content between yellow and brown soybean cultivars adapted in Canada. Moreover, Redondo-Cuenca et al. [32] found significant variations in protein and fat contents between ecological and transgenic yellow and green soybeans of different countries of origin. Again, the differences in genotypes, growing conditions, and year of cultivation could be the causes for such variations [20,27].

In general, our results signify that seed coat color alone might not provide useful information regarding the relative protein and oil contents in yellow, light-yellow, and greenish-yellow soybeans. Besides, the significant variation of total protein and total oil content observed between the individual soybeans was likely the result of genetic differences because the soybeans were cultivated under similar environmental conditions. This suggests that soybeans of different seed coat colors should be evaluated individually for their use in soy–protein formulations and oil production.

### 3.2. Fatty Acid Contents

Table 2 shows the contents of individual and total fatty acids for each of the soybean accessions. Among the saturated fatty acids, palmitic acid (9.90–12.19%) was dominant over stearic acid (2.45–3.60%) in all the soybeans and previous studies also documented a similar trend [29,33,34]. A recent genome-wide association study specified that several genes control the levels of stearic acid and palmitic acid in soybeans, and directed that such variation could favor one over the other during their biosynthesis [35]. Accessions YL10 and YL5 had the highest palmitic acid and stearic acid contents, respectively, each differing significantly from the rest of the accessions (*p* < 0.05) (Table 2). Taekwangkong, the control cultivar, presented the lowest palmitic acid content which differed significantly from the rest of the accessions except GY13, GY15, and LY16 (*p* < 0.05). Likewise, the stearic acid content in taekwangkong was much lower than the rest accessions except LY18, LY17, and LY16. These latter three accessions contained the first, second, and third-lowest levels of stearic acid in their order. An elevated level of stearic acid is desired in soybean seeds owing to its potential to replace *trans*-fatty acids and reduce the risk of cardiovascular diseases [27]. In this regard, the soybean accessions such as YL2, YL5, and GY12 which contained a relatively higher level of stearic acid in their seeds could be important resources.

Concerning unsaturated fatty acids, the contents of oleic acid, linoleic acid, and linolenic acid were in the ranges of 16.02–38.74, 43.22–59.15, and 5.37–10.35% with means of 25.35, 52.86, and 7.86%, respectively (Table 2). These ranges were comparable with those reported by Kim et al. [9] but slightly wider than those reported by Shin et al. [34]. In other studies, a less wide range of oleic acid and more wide range of linoleic acid and linolenic acid were reported [29,36]. In addition to differences in the genotype, discrepancies in the number of studied soybean genetic resources could cause such variations. The level of linoleic acid was dominant in every accession followed by the level of oleic acid. Previous studies also noted similar results and outlined that the desaturation of oleic acid to linoleic acid by FAD2 and FAD3 enzymes during fatty acid biosynthesis could be the cause for such a trend [9,29,33,34,37]. Oleic acid exhibited the highest coefficient of variation (CV) (29.03%) demonstrating a high variability between the soybeans, whereas linolenic acid displayed the lowest CV (1.72%). Among the studied accessions, linoleic acid and linolenic acid contents were the highest in accessions GY11 and YL2, the former differing significantly from all but accessions YL2, YL5, and YL6, while the latter differing significantly from all the other accessions (*p* < 0.05). Unlike the levels of palmitic acid and stearic acid, the control cultivar was rich in its level of oleic acid. Except for accessions LY16 and LY18, the rest of the accessions contained a much lower level of oleic acid than the control cultivar (*p* < 0.05). The control cultivar also had the third-lowest level of linoleic acid next to accessions LY18 and LY16, and the fourth-lowest level of linolenic acid after accessions LY16, LY18, and LY19 (*p* < 0.05). The oleic acid level in taekwangkong found in this study was much higher, while the linoleic acid level was slightly lower than those reported by Cho et al. [4] almost a decade ago for the same cultivar. The difference in cultivation year could be the major cause for such variation. Oxidative instability characterizes linoleic acid, a polyunsaturated omega-3 fatty acid, and causes rancidity and reduced shelf-life of soybean oil and soy products. Because of this, attempts have been made to reduce the level of linoleic acid in soybeans through breeding. Together, increasing the level of oleic acid is commonly considered owing to its health benefits and its role to improve oil oxidative stability [28,36,38,39]. The fact that taekwangkong presented a higher level of oleic acid and a lower level of linoleic acid than most of the other accessions infers its significance as a commercial cultivar. In this regard, accessions LY16 and LY18, which were found to contain a higher level of oleic acid and a lower level of linoleic acid than the control cultivar, could be potential resources for the production of good quality soybean oil. In general, the soybean accessions exhibited a higher level of TUFA than TSFA in their seeds (Table 2). The TUFA level in the control cultivar was significantly higher while the TSFA level was significantly lower than the rest of the accessions except for LY16, GY13, and GY15 (*p* < 0.05).

The influence of seed coat color on the levels of individual and total fatty acids was also similarly analyzed. By comparison, light-yellow soybeans had a high level of oleic acid and low levels of stearic acid, linoleic acid, and linolenic acid; each, except linoleic acid, differing significantly from the other two groups (*p* < 0.05) (Figure 2, Table S1). Yellow soybeans displayed high levels of palmitic acid, stearic acid, and linolenic acid, and a low level of oleic acid. Yellow soybeans studied in this work contained a lower average oleic acid but higher average linoleic acid and linolenic acid levels than those reported by Slavin et al. [33] and Shin et al. [34]. The highest linoleic acid and the lowest palmitic acid levels were each found in greenish-yellow soybeans. These observations further attest to the contrasting relationship between oleic acid and linoleic acid regardless of the difference in seed coat color [26]. It is notable from Figure 2 that the variations of palmitic acid, linoleic acid, TSFA, and TUFA were not significantly different between the three groups of soybeans (*p* < 0.05). Limited studies were available regarding the influence of seed coat color on the levels of fatty acids and the reported data were inconsistent. For instance, Cho et al. [4], Lee et al. [9], and Slavin et al. [33] separately investigated the variation of these five fatty acids between yellow, black, brown, and green soybeans. The former study failed to note significant variations while the latter two reported significant variations of individual fatty acids in response to seed coat color. Generally, our results suggest that light-yellow soybeans could be important sources of good quality soybean oil owing to the presence of a simultaneously high level of oleic acid and a low level of linoleic acid in their seeds. Besides, they could be suitable candidates to develop soybean cultivars with altered unsaturated fatty acids through breeding [28,38,39].

### 3.3. Total Phenolic Content and Antioxidant Activities

**Figure 3:**
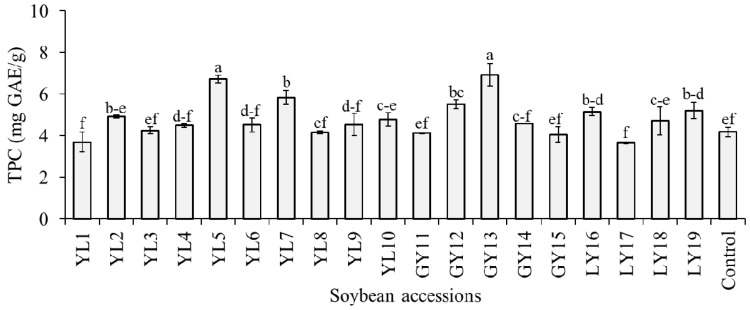
Total phenolic content (TPC) of individual soybeans. Bars with different superscript letters (a–f) represent significantly different values (*p* < 0.05).

The level of TPC in the whole seeds of the studied soybeans was estimated as mg GAE/g of dried seed weight and the result is presented in Figure 3. With an average of 4.81 mg GAE/g, the TPC was in the range of 3.65–6.92 mg GAE/g, where most values gathered between 4.07 and 4.93 mg GAE/g. The highest TPC was found in accession GY13 and this value was significantly different from the rest of the accessions except YL5 (*p* < 0.05). Previously, several studies estimated the level of TPC in seeds of various soybean genotypes. Different researchers used varied sorts of extraction protocols and sample preparation techniques, and, hence, the reported TPC values were wide-ranging [16,18,33,40]. In general, the TPC range found in this study was within these previously reported TPC values for soybean seeds. It is notable from Figure 3 that among the 19 accessions, only 7 had a significantly high level of TPC than the control cultivar (*p* < 0.05). The TPC level in taekwangkong (4.18 ± 0.24 mg GAE/g) found in this study was close to the TPC level reported by Ku et al. (~3.89 GAE/g) [41] and Lee et al. (3.70 mg GAE/g) [42] for the same cultivar. In a later study, Eum et al. [43] showed that germination of this cultivar for 3 to 7 days in the dark could help to enhance its TPC level. 

**Figure 4:**
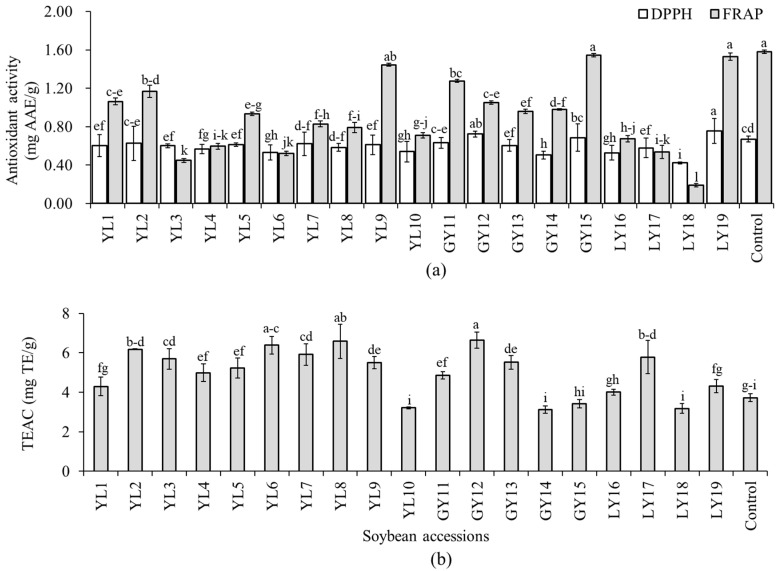
Antioxidant activities of individual soybeans. DPPH-radical scavenging activity and ferric reducing antioxidant power (**a**) and Trolox equivalent antioxidant capacity (**b**). Bars with different superscript letters (a–i) represent significantly different values (*p* < 0.05).

Several in vitro assays are commonly used to estimate the antioxidant properties of soybeans and other foods. Because of the specificity and sensitivity of these methods, however, a single assay does not always provide a complete examination of antioxidant potentials of extracted phenolic compounds [16]. In this study, the antioxidant activity of each soybean was determined in terms of three assays including DPPH-radical scavenging activity, TEAC, and FRAP to get a broad perspective on their properties. The DPPH-radical scavenging activity, TEAC, and FRAP were in the ranges of 0.42–0.76 mg AAE/g, 3.13–6.64 mg TE/g, and 0.19–1.58 mg AAE/g, respectively (Figure 4). Relatively, wide variation among the soybean accessions was found in FRAP (CV: 42.14%). Accession LY19 showed the highest DPPH-radical scavenging activity which differed significantly from all but accession GY12. Only these two accessions, LY19 and GY12, showed a significantly high DPPH-radical scavenging activity than the control cultivar (*p* < 0.05). Accession GY12 also displayed the highest TEAC activity which differed significantly from all the other accessions except YL8 and YL6. Accession GY15 had the second-highest FRAP activity next to the control cultivar.

The influence of seed coat color was once again investigated. The average TPC decreased in the order of greenish-yellow (5.04 mg GAE/g) > yellow (4.80 mg GAE/g) > light-yellow (4.69 mg GAE/g) soybeans (Figure 2, Table S1). However, statistical analysis did not show any significant difference between the colored soybeans (*p* < 0.05). The average TPC level in yellow, light-yellow, and greenish-yellow soybeans found in this study was comparable with those values found in Indonesian soybeans of similar seed coat colors [44]. This previous study also did not observe a significant variation of TPC between yellow, light-yellow, and greenish-yellow soybeans which agreed with our results. Besides, the TPC range in yellow soybeans found in this study (3.70–6.74 mg GAE/g) was slightly higher than a previously reported value (3.0–4.5 mg GAE/g) determined using six yellow soybean genotypes [45,46]. Greenish-yellow soybeans, which had the highest average TPC level, showed the highest average DPPH-radical scavenging activity than FRAP, and hence, could be good sources of antioxidants. On the other hand, light-yellow soybeans that had the lowest average TPC level showed the lowest activities in all the antioxidant activities. These observations were congruent with previous findings where a high level of TPC was highly associated with a pronounced antioxidant activity [16,18,33]. By comparison, the average TEAC activity was exclusively high in yellow soybeans than in light-yellow and greenish-yellow soybeans. Despite these variations, however, none of the antioxidant activities were significantly different between the colored soybeans (*p* < 0.05).

Previous studies reported varying results regarding the relative level of TPC in colored soybeans. For instance, Kumar et al. [47] did not find a significant variation in the level of TPC between yellow, black, and green soybeans while Xu and Chang [16] found a significant variation between yellow and black soybeans. In another study, a significant variation was noted in the level of TPC among various colored soybeans but not between individual soybean genotypes [18]. Many of these studies also attested and estimated the antioxidant capacities of colored soybeans. However, differences in assays, concentrations, protocols and reporting methods made it difficult to compare with our findings. In many of these previous studies, black soybeans tend to repetitively outweigh other colored soybeans in their TPC level and antioxidant potentials [16,33,40]. Nevertheless, it is worth noting that there were also occasions in which other colored soybeans, such as yellow and green, contained a higher level of TPC than black soybeans [47,48]. Hence, molecular level investigations are highly recommended to drive a concluding remark.

### 3.4. Principal Component, Hierarchical Cluster and Correlation Analysis

**Figure 5:**
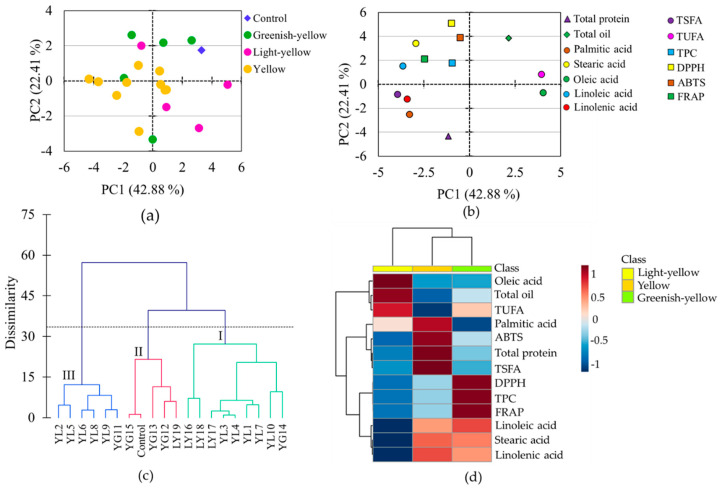
Score plot of soybeans (**a**) and loading plot of variables (**b**) from the principal component analysis, Ward’s cluster analysis (**c**), and heatmap showing the variation of metabolites among soybeans according to seed coat color (**d**). TEAC: Trolox equivalent antioxidant capacity; DPPH: DPPH-radical scavenging activity; FRAP: Ferric reducing antioxidant power; GY: Greenish-yellow; LY: Light-yellow; TPC: Total phenolic content; TSFA: total saturated fatty acid; TUFA: Total unsaturated fatty acid; YL: Yellow.

Several chemometric tools, combined with metabolomics data, are used to view the association between variables and the difference between studied samples. Besides, they are applicable to pinpoint variables that contribute most to the observed differences between samples [49]. In line with these, PCA, cluster, and heatmap analyses were conducted using the whole data set. The PCA provided 4 components that had eigenvalues > 1 and contributed 84.82% of the total variability (Table 3). Among these, the first two components accounted for 42.88% (PC1) and 22.41% (PC2) of the cumulative variance, and hence, the score plot (Figure 5a), and loading plot (Figure 5b) obtained over these two components were analyzed to see the distribution and association of metabolite contents, antioxidant activities, and the soybean accessions. It is notable from the score plot that all light-yellow soybeans except one were distributed along the positive side of PC1, whereas all greenish-yellow soybeans except one were distributed along the positive side of PC2 (Figure 5a). Except for stearic acid, the rest of the fatty acids were the foremost contributors for the variability observed along PC1, oleic acid (15.36%) followed by linoleic acid (12.53%) being the most discriminating parameters (Figure 5b, Table 3). The other variables had substantial contributions along PC2, DPPH-radical scavenging activity (24.44%) and total protein content (17.86%) being the two dominant parameters. 

The grouping of the soybeans observed in the PCA was further confirmed by cluster analysis which grouped the soybeans into three major classes each having two major subgroups. Group I and group II contained nine and six accessions, respectively, while group III contained five soybeans including the control cultivar. It is clear from Figure 5c that three out of the four light-yellow soybeans were closely clustered in group I, while three out of the five greenish-yellow soybeans were clustered in group II, which was congruent with the PCA results. In general, the PCA and cluster analysis revealed a relatively clear association of light-yellow soybeans and greenish-yellow soybeans though they were not firmly separated from those of yellow soybeans (Figure 5a,c).

The relative associations between metabolites and antioxidant activities were visible in the loading plot (Figure 5b). Oleic acid was highly associated with the total oil and least associated with the other fatty acids. In the Pearson’s correlation analysis oleic acid was also positively correlated with the total oil content and negatively correlated with the rest of the fatty acids at various significance levels (Table S2). In particular, the correlations of oleic acid with linoleic acid (*r* = −0.969) and linolenic acid (*r* = −0.780) were each significant (*p* < 0.0001). These observations were in agreement with several previous findings [9,50]. The negative relationship between total oil and total protein contents and the positive relationship between TPC and antioxidant activities were also evident from both the loading plot and correlation analysis. It is interesting to note that none of the fatty acids displayed a significant association with total protein (Table S2) which was consistent with a previous report [9]. Overall, the pair-wise correlations between the metabolites observed in the Pearson’s correlation analysis were in agreement with the associations of the metabolites observed in the loading plot of the PCA (Figure 5b, Table S2).

## 4. Conclusions

In this study, we cultivated seeds of 19 soybean accessions and a reference cultivar (taekwangkong) in Korea and grouped them as yellow, greenish-yellow, and light-yellow based on their seed coat colors. Then, we comprehensively analyzed the contents of total protein, total oil, total phenolic, palmitic acid, stearic acid, oleic acid, linoleic acid, and linolenic acid, and antioxidant activities including DPPH-radical scavenging activity, Trolox equivalent antioxidant capacity, and ferric reducing antioxidant potential using extracts of their whole seeds. The accessions showed wide variability in their metabolite contents as well as antioxidant activities. By comparison, greenish-yellow soybeans had high levels of total phenolic content and pronounced antioxidant properties than yellow and light-yellow soybeans. On the other hand, light-yellow soybeans were characterized by a high level of oleic acid and low levels of linoleic acid and linolenic acid, while yellow soybeans had high levels of TSFA and total protein (Figure 5d). In particular, accessions LY16 and LY18 had a higher level of oleic acid and lower levels of linoleic acid and linolenic acid, even than the control cultivar. The simultaneous presence of a high level of oleic acid and a low level of linoleic acid in soybean seeds is the desired characteristic and is preferred during breeding. Hence, these latter two accessions (LY16 and LY18) could be potential resources and used for the production of good quality soybean oil. Overall, this study could provide a useful background to consumers and food industries regarding the assessment of soybeans of different seed coat colors in terms of metabolite contents, nutritional qualities, and biological activities. Moreover, the soybean accessions with distinct characteristics and nutritional contents identified in this study could be important resources for consumption and cultivar development.

## Figures and Tables

**Table 1:** Total protein and total oil contents in seeds of individual soybeans.

Name	Introduction Number	Code	Total Protein (%)	Total Oil (%)
Yellow				
YJ208-1	024099	YL1	41.63 ± 0.16 ^b^	16.75 ± 0.27 ^hi^
Kongnamul-kong	113218	YL2	39.60 ± 0.21 ^e^	15.97 ± 0.29 ^jk^
Nongrim51ho	155963	YL3	38.38 ± 0.06 ^h^	17.18 ± 0.08 ^f–h^
PI467319	171080	YL4	39.21 ± 0.15 ^f^	17.16 ± 0.18 ^gh^
Hoseo	229421	YL5	41.34 ± 0.23 ^c^	15.48 ± 0.20 ^kl^
Kongnamul-kong	231360	YL6	39.31 ± 0.11 ^ef^	16.81 ± 0.30 ^hi^
Shinpaldalkong2ho	263155	YL7	40.37 ± 0.12 ^d^	18.06 ± 0.16 ^c–e^
Uram	263167	YL8	39.31 ± 0.09 ^ef^	17.26 ± 0.48 ^f–h^
GNU-2007-14613	274571	YL9	36.46 ± 0.05 ^k^	15.63 ± 0.21 ^kl^
Himeyudaga	156272	YL10	40.61 ± 0.11 ^d^	15.17 ± 0.32 ^l^
Greenish-yellow				
Kongnamul-kong	104690	GY11	38.84 ± 0.08 ^g^	15.40 ± 0.39 ^kl^
KLS87248	153844	GY12	37.99 ± 0.04 ^i^	18.98 ± 0.12 ^a^
Myeongjunamul-kong	219581	GY13	37.92 ± 0.05 ^i^	18.27 ± 0.16 ^b–d^
Jungmo3008ho	270002	GY14	44.19 ± 0.08 ^a^	13.45 ± 0.21 ^m^
GNU-2007-14723	274592	GY15	36.28 ± 0.14 ^k^	18.77 ± 0.22 ^ab^
Light-yellow				
Jangkong	195514	LY16	38.21 ± 0.20 ^hi^	18.46 ± 0.36 ^a–c^
Chungnamyeongi-1997-3	263852	LY17	40.32 ± 0.20 ^d^	16.37 ± 0.09 ^ij^
Duruol	269982	LY18	39.03 ± 0.28 ^fg^	17.91 ± 0.25 ^c–e^
CS00728	324099	LY19	37.60 ± 0.15 ^j^	17.63 ± 0.33 ^e–g^
Taekwangkong (Control)			37.36 ± 0.04 ^j^	17.78 ± 0.44 ^d–f^
Range (%)			36.28–44.19	13.45–18.98
Total mean (%)			39.20	16.92
CV(%)			4.79	8.36

Values in each column with different superscript letters (a–m) are significantly different (*p* < 0.05).

**Table 2:** Contents (%) of individual and total fatty acids in seeds of the studied soybeans.

Accession	Palmitic Acid	Stearic Acid	Oleic Acid	Linoleic Acid	Linolenic Acid	TSFA ^1^	TUFA ^2^
Yellow							
YL1	10.54 ± 0.14 ^f^	3.17 ± 0.05 ^de^	28.23 ± 0.09 ^cd^	50.71 ± 0.14 ^gh^	7.34 ± 0.05 ^g^	13.72 ± 0.18 ^de^	86.28 ± 0.18 ^de^
YL2	11.81 ± 0.07 ^b^	3.36 ± 0.03 ^bc^	16.02 ± 0.06 ^j^	58.46 ± 0.11 ^a^	10.35 ± 0.06 ^a^	15.17 ± 0.09 ^a^	84.83 ± 0.09 ^h^
YL3	10.81 ± 0.69 ^ef^	2.92 ± 0.10 ^hi^	25.68 ± 0.38 ^d–f^	53.26 ± 0.80 ^d–f^	7.32 ± 0.41 ^g^	13.74 ± 0.79 ^de^	86.26 ± 0.79 ^de^
YL4	10.54 ± 0.07 ^f^	2.92 ± 0.01 ^hi^	25.84 ± 0.03 ^c–f^	52.53 ± 0.04 ^d–g^	8.18 ± 0.01 ^f^	13.45 ± 0.07 ^ef^	86.55 ± 0.07 ^cd^
YL5	11.52 ± 0.15 ^c^	3.60 ± 0.02 ^a^	19.26 ± 0.16 ^i^	57.10 ± 0.11 ^ab^	8.52 ± 0.11 ^ef^	15.12 ± 0.16 ^a^	84.88 ± 0.16 ^h^
YL6	11.51 ± 0.03 ^c^	3.05 ± 0.03 ^e–h^	17.56 ± 0.06 ^ij^	59.05 ± 0.08 ^a^	8.83 ± 0.03 ^de^	14.56 ± 0.03 ^bc^	85.44 ± 0.03 ^fg^
YL7	10.74 ± 0.04 ^f^	3.13 ± 0.03 ^de^	28.33 ± 0.11 ^c^	50.91 ± 0.07 ^gh^	6.90 ± 0.05 ^gh^	13.87 ± 0.05 ^d^	86.13 ± 0.05 ^e^
YL8	11.24 ± 0.05 ^cd^	3.32 ± 0.04 ^c^	23.36 ± 0.11 ^f–h^	52.91 ± 0.12 ^d–g^	9.16 ± 0.06 ^cd^	14.56 ± 0.08 ^bc^	85.44 ± 0.08 ^fg^
YL9	10.82 ± 0.02 ^ef^	3.09 ± 0.01 ^ef^	22.22 ± 0.04 ^h^	54.33 ± 0.06 ^cd^	9.54 ± 0.06 ^bc^	13.91 ± 0.02 ^d^	86.09 ± 0.02 ^e^
YL10	12.19 ± 0.02 ^a^	2.80 ± 0.03 ^ij^	27.71 ± 0.15 ^c–e^	49.22 ± 0.12 ^hi^	8.08 ± 0.06 ^f^	14.98 ± 0.04 ^a^	85.02 ± 0.04 ^h^
Greenish-yellow							
GY11	11.19 ± 0.02 ^d^	3.07 ± 0.01 ^e–g^	18.11 ± 0.05 ^ij^	59.15 ± 0.05 ^a^	8.48 ± 0.04 ^ef^	14.26 ± 0.02 ^c^	85.74 ± 0.02 ^f^
GY12	10.84 ± 0.13 ^ef^	3.46 ± 0.31 ^b^	22.90 ± 6.19 ^gh^	54.44 ± 5.37 ^cd^	8.35 ± 1.00 ^f^	14.31 ± 0.18 ^bc^	85.69 ± 0.18 ^fg^
GY13	9.90 ± 0.03 ^g^	3.25 ± 0.01 ^cd^	23.31 ± 0.13 ^f–h^	56.32 ± 0.09 ^bc^	7.22 ± 0.02 ^g^	13.15 ± 0.03 ^fg^	86.85 ± 0.03 ^bc^
GY14	10.78 ± 0.09 ^ef^	2.82 ± 0.02 ^ij^	25.34 ± 0.16 ^e–g^	51.43 ± 0.18 ^fg^	9.63 ± 0.09 ^b^	13.60 ± 0.11 ^de^	86.40 ± 0.11 ^de^
GY15	10.02 ± 0.04 ^g^	2.96 ± 0.03 ^f–h^	28.22 ± 0.03 ^cd^	51.79 ± 0.08 ^e–g^	7.00 ± 0.01 ^gh^	12.99 ± 0.07 ^gh^	87.01 ± 0.07 ^ab^
Light-yellow							
LY16	9.94 ± 0.07 ^g^	2.73 ± 0.04 ^j^	38.74 ± 0.49 ^a^	43.22 ± 0.43 ^j^	5.37 ± 0.07 ^j^	12.67 ± 0.10 ^h^	87.33 ± 0.10 ^a^
LY17	10.85 ± 0.02 ^ef^	2.57 ± 0.01 ^k^	26.13 ± 0.05 ^c–e^	52.30 ± 0.03 ^d–g^	8.15 ± 0.04 ^f^	13.42 ± 0.02 ^ef^	86.58 ± 0.02 ^cd^
LY18	11.10 ± 0.02 ^de^	2.45 ± 0.00 ^k^	32.60 ± 0.15 ^b^	47.81 ± 0.13 ^i^	6.04 ± 0.04 ^i^	13.55 ± 0.02 ^de^	86.45 ± 0.02 ^de^
LY19	11.49 ± 0.04 ^c^	3.15 ± 0.01 ^de^	25.33 ± 0.14 ^e–g^	53.86 ± 0.06 ^de^	6.18 ± 0.03 ^i^	14.63 ± 0.06 ^b^	85.37 ± 0.06 ^g^
Taekwangkong	9.88 ± 0.04 ^g^	2.94 ± 0.01 ^g–i^	32.18 ± 0.04 ^b^	48.38 ± 0.04 ^i^	6.62 ± 0.05 ^h^	12.82 ± 0.02 ^gh^	87.18 ± 0.01 ^ab^
Range (%)	9.90–12.19	2.45–3.60	16.02–38.74	43.22–59.15	5.37–10.35	12.67–15.17	84.83–87.33
Mean (%)	10.89	3.04	25.35	52.86	7.86	13.92	86.08
CV(%)	5.91	9.49	21.63	7.63	16.66	5.40	0.87

^1^ Total saturated fatty acid; ^2^ Total unsaturated fatty acid. Values in each column with different superscript letters (a–k) are significantly different (*p* < 0.05).

**Table 3:** Contribution (%) of variables to the first four principal components.

Variables	PC1	PC2	PC3	PC4
Total protein	1.24	17.86	0.01	1.70
Total oil	4.35	13.91	15.57	0.27
Palmitic acid	10.20	5.99	0.52	6.72
Stearic acid	8.21	11.07	0.95	1.76
Oleic acid	15.36	0.47	0.51	5.83
Linoleic acid	12.53	2.17	0.13	7.32
Linolenic acid	11.01	1.41	6.03	11.19
TPC ^1^	0.84	2.98	24.89	15.01
DPPH ^2^	0.92	24.44	5.07	3.22
TEAC ^3^	5.75	4.18	12.03	24.88
FRAP ^4^	0.25	14.17	32.34	7.24
TSFA ^5^	14.68	0.67	0.98	7.43
TUFA ^6^	14.68	0.67	0.98	7.43
Eigenvalue	5.57	2.91	1.41	1.13
Variability (%)	42.88	22.41	10.81	8.72
Cumulative (%)	42.88	65.29	76.10	84.82

^1^ Total phenolic content; ^2^ DPPH-radical scavenging activity; ^3^ Trolox equivalent antioxidant capacity; ^4^ Ferric reducing antioxidant power; ^5^ Total saturated fatty acid; ^6^ Total unsaturated fatty acid.

## Data Availability

Data is contained within the article or Supplementary Materials.

## Supplementary Materials

The following are available online at https://www.mdpi.com/article/10.3390/antiox10081210/s1, Table S1: Ranges, means, and coefficients of variation of all metabolites and antioxidant activities of soybean accessions according to the seed coat color, Table S2: Pair-wise correlation between metabolites and antioxidant activities computed using Pearson’s analysis. Values in the lower bound of the table represent correlation coefficient (r) and those in the upper bound represent the corresponding *p*-values.
