# Supplementary material for: Metabolite Contents and Antioxidant Activities of Soybean (Glycine max (L.) Merrill) Seeds of Different Seed Coat Colors
Source: Antioxidants (Basel). 2021 Jul 28;10(8):1210. doi: 10.3390/antiox10081210 (PMC8388989; doi:10.3390/antiox10081210)
Supplement: Supplementary file 1 [file antioxidants-10-01210-s001.zip › antioxidants-1283272 -Supplementary.pdf]

Supplementary materials

Table S1. Ranges, means, and coefficients of variation of all metabolites and antioxidant activities of soybean accessions according to the seed coat color.

Metabolites/ antioxidant activities	Values	Seed coat color		
		YL[7]	GY[8]	LY[9]
Total protein	Range (%)	36.46-4163	36.28-44.19	37.60-40.32
	Mean (%)	39.62[a]	39.04[a]	38.79[a]
	CV (%)	3.80	7.74	3.04
Total oil	Range (%)	15.17-18.06	13.45-18.98	16.37-18.46
	Mean (%)	16.55[a]	16.97[a]	17.59[a]
	CV (%)	5.66	14.38	5.05
Palmitic acid	Range (%)	10.54-12.19	9.90-11.19	9.94-11.49
	Mean (%)	11.17[a]	10.55[a]	10.84[a]
	CV (%)	5.09	5.29	6.05
Stearic acid	Range (%)	2.80-3.60	2.82-3.46	2.45-3.15
	Mean (%)	3.14[a]	3.11[a]	2.72[b]
	CV (%)	7.67	8.09	11.14
Oleic acid	Range (%)	16.02-28.33	18.11-28.22	25.33-38.74
	Mean (%)	23.42[b]	23.58[b]	30.70[a]
	CV (%)	19.33	15.74	20.43
Linoleic acid	Range (%)	49.22-59.05	51.43-59.15	43.22-53.86
	Mean (%)	53.85[a]	54.63[a]	49.30[a]
	CV (%)	6.25	5.91	9.73
Linolenic acid	Range (%)	6.90-10.35	7.00-9.63	5.37-8.15
	Mean (%)	8.42[a]	8.14[a]	6.44[b]
	CV (%)	12.87	13.07	18.61
TSFA[1]	Range (%)	13.45-15.17	12.99-14.31	12.67-14.63
	Mean (%)	14.31[a]	13.66[a]	13.57[a]
	CV (%)	4.50	4.47	5.95
TUFA[2]	Range (%)	84.83-86.55	85.69-87.01	85.37-87.33
	Mean (%)	85.69[a]	86.34[a]	86.43[a]
	CV (%)	0.75	0.71	0.93
TPC[3]	Range (mg GAE/g)	3.70-6.74	4.07-6.92	3.65-5.22
	Mean (mg GAE/g)	4.80[a]	5.04[a]	4.69[a]
	CV (%)	18.38	23.79	15.55
DPPH[4]	Range (mg AAE/g)	0.53-0.63	0.50-0.72	0.42-0.76
	Mean (mg AAE/g)	0.59[a]	0.63[a]	0.57[a]
	CV	5.61	13.33	24.40
TEAC[5]	Range (mg TE/g)	3.21-6.58	3.13-6.64	3.17-5.78
	Mean (mg TE/g)	5.40[a]	4.71[a]	4.32[a]
	CV	19.08	31.07	25.17
FRAP[6]	Range (mg AAE/g)	0.45-1.44	0.96-1.54	0.19-1.53
	Mean (mg AAE/g)	0.85[a]	1.16[a]	0.73[a]
	CV	36.40	21.37	77.65

Mean values in a row with different superscript letters are significantly different ($p < 0.05$). [1]Total saturated fatty acid; [2]Total unsaturated fatty acid; [3]Total phenolic content; [4]DPPH-radical scavenging activity; [5]Trolox equivalent antioxidant capacity; [6] Ferric reducing antioxidant power; [7]Yellow; [8]Greenish-yellow; [9]Light-yellow.

Table S2. Pair-wise correlation between metabolites and antioxidant activities computed using Pearson's analysis. Values in the lower bound of the table represent correlation coefficient (*r*) and those in the upper bound represent the corresponding *p*-values.

Variables	TPC	DPPH	TEAC	FRAP	Total protein	Total oil	Palmitic acid	Stearic acid	Oleic acid	Linoleic acid	Linolenic acid	TSFA	TUFA
TPC[1]	1	0.518	0.537	0.786	0.903	0.429	0.861	0.033	0.671	0.513	0.547	0.510	0.510
DPPH[2]	0.158	1	0.149	0.001	0.065	0.206	0.910	0.004	0.159	0.086	0.936	0.339	0.339
TEAC[3]	0.151	0.344	1	0.847	0.426	0.397	0.661	0.011	0.011	0.010	0.077	0.174	0.174
FRAP[4]	0.067	0.750	-0.048	1	0.201	0.792	0.796	0.030	0.189	0.162	0.427	0.543	0.543
Total protein	-0.030	-0.432	-0.194	-0.307	1	0.003	0.239	0.822	0.842	0.767	0.207	0.371	0.371
Total oil	0.193	0.304	0.206	-0.065	-0.646	1	0.028	0.850	0.086	0.285	0.003	0.084	0.084
Palmitic acid	-0.043	-0.028	0.108	-0.064	0.284	-0.503	1	0.487	0.030	0.130	0.058	< 0.0001	< 0.0001
Stearic acid	0.491	0.625	0.571	0.498	-0.055	0.047	0.170	1	0.004	0.005	0.096	0.014	0.014
Oleic acid	-0.104	-0.336	-0.571	-0.315	-0.049	0.405	-0.498	-0.626	1	< 0.0001	< 0.0001	0.001	0.001
Linoleic acid	0.160	0.405	0.574	0.334	-0.073	-0.258	0.360	0.615	-0.969	1	0.004	0.013	0.013
Linolenic acid	-0.147	0.020	0.415	0.193	0.303	-0.650	0.443	0.393	-0.780	0.626	1	0.018	0.018
TSFA[5]	0.161	0.232	0.325	0.149	0.218	-0.406	0.915	0.553	-0.678	0.556	0.536	1	< 0.0001
TUFA[6]	-0.161	-0.232	-0.325	-0.149	-0.218	0.406	-0.915	-0.553	0.678	-0.556	-0.536	-1.000	1

[1]Total phenolic content; [2]DPPH-radical scavenging activity; [3]Trolox equivalent antioxidant capacity; [4]Ferric reducing antioxidant power; [5]Total saturated fatty acid; [6]Total unsaturated fatty acid.
